# Current concepts in the diagnosis and management of antiphospholipid syndrome and ocular manifestations

**DOI:** 10.1186/s12348-021-00240-8

**Published:** 2021-04-09

**Authors:** Gunay Uludag, Neil Onghanseng, Anh N. T. Tran, Muhammad Hassan, Muhammad Sohail Halim, Yasir J. Sepah, Diana V. Do, Quan Dong Nguyen

**Affiliations:** 1 Spencer Center for Vision Research, Byers Eye Institute, Stanford University, 2370 Watson Court, Suite 200, Palo Alto, CA 94303 USA; 2 Ocular Imaging Research and Reading Center, Sunnyvale, CA USA

**Keywords:** Antiphospholipid syndrome, Antiphospholipid antibodies, Ocular manifestations

## Abstract

Antiphospholipid syndrome (APS) is an autoimmune disorder associated with obstetrical complications, thrombotic complications involving both arteries and veins, and non-thrombotic manifestations affecting multiple other systems presenting in various clinical forms. Diagnosis requires the presence of antiphospholipid antibodies. The exact pathogenesis of APS is not fully known. However, it has recently been shown that activation of different types of cells by antiphospholipid antibodies plays an important role in thrombosis formation. Ocular involvement is one of the important clinical manifestations of APS and can vary in presentations. Therefore, as an ophthalmologist, it is crucial to be familiar with the ocular findings of APS to prevent further complications that can develop. Furthermore, the ongoing identification of new and specific factors contributing to the pathogenesis of APS may provide new therapeutic options in the management of the disease in the future.

## Background

Antiphospholipid syndrome (APS) is a systemic autoimmune condition characterized by vascular thrombosis involving both arteries and veins, fetal losses, and thrombocytopenia in the presence of antiphospholipid antibodies (aPL) including lupus anticoagulant (LA), anti-cardiolipin antibodies (aCL), and anti-β2 glycoprotein-I (anti-β2GPI) [1, 2].

Antiphospholipid syndrome can be divided into two forms: primary and secondary. Patients with no clinical or laboratory evidence of any other associated systemic disease are defined as having primary APS. Secondary APS is defined as the patient having other comorbid conditions, most commonly systemic lupus erythematosus (SLE). Association with other autoimmune diseases, drug reactions, infections, and malignancies are also under the classification of secondary APS [1, 3].

Clinical manifestations of APS fall under a wide spectrum including asymptomatic carrier patients with aPL positivity, classical APS with vascular thrombosis and/or fetal losses, aPL positivity without thrombotic APS findings (i.e., thrombocytopenia, hemolytic anemia, livedo reticularis, and seizures), or catastrophic APS characterized by multi-organ failure due to multiple microthrombosis [1, 4, 5].

## Epidemiology

Antiphospholipid syndrome typically affects young to middle-aged adults, most commonly between the ages of 15 and 50. Both primary and secondary APS are more common in women than men in about a 1:3.5 male-to-female ratio for primary APS and 1:7 for secondary APS associated with SLE [6]. The estimated incidence of APS is around 5 new cases per 100,000 persons per year, with a prevalence of around 40–50 cases per 100,000 persons [1]. APL positivity was reported as 13.5% for stroke, 11% for myocardial infarction, 9.5% with deep venous thrombosis, and 6% in pregnancy mortality [7].

Ocular findings are seen in 15–88% of patients with primary APS [3]. Taking into account the higher frequency of ocular findings of APS, regular consult with an ophthalmologist may be able to detect early signs leading to a diagnosis of APS and may prevent life-threatening conditions associated with systemic thrombosis.

## Diagnosis

A diagnosis of APS is based on the revised Sapporo criteria and requires the presence of at least one clinical criteria (vascular thrombosis and/or pregnancy morbidity) and one laboratory criteria (persistence of at least 12 weeks of lupus anticoagulant and/or medium-high titers of IgG or IgM autoantibodies against β2GPI or cardiolipin) [8] (Table 1).

**Table 1:** Revised Sapporo criteria for the antiphospholipid syndrome (APS) [8]

**Clinical Criteria**	
**1. Vascular thrombosis:** One or more presence of arterial, venous, or small vessel thrombosis in any tissue or organ. Thrombosis must be verified by objective validated criteria, i.e., unequivocal findings of appropriate imaging studies or histopathology. For histologic confirmation, thrombosis should be present without remarkable inflammation in the vessel wall.	
**2. Pregnancy morbidity:**	
• One or more unexplained deaths of a morphologically normal fetus at or beyond the 10th week of gestation, with normal fetal morphology documented by ultrasound or by direct examination of the fetus.	
• One or more premature births of a morphologically normal neonate before the 34th week of gestation because of: eclampsia or severe preeclampsia defined according to standard definitions or recognized features of placental insufficiency.	
• Three or more unexplained consecutive spontaneous abortions before the 10th week of gestation, with maternal anatomic or hormonal abnormalities and paternal and maternal chromosomal causes excluded.	
In studies of populations of patients who have more than one type of pregnancy morbidity, investigators are strongly encouraged to stratify groups of subjects according to one of the three criteria above.	
**Laboratory Criteria:**	
**1.** Lupus anticoagulant (LA) present in plasma, on two or more occasions at least 12 weeks apart, detected according to the guidelines of the International Society on Thrombosis and Haemostasis (Scientific Subcommittee on LAs/phospholipid-dependent antibodies).	
**2.** Anticardiolipin (aCL) antibody of IgG and/or IgM isotype in serum or plasma, present in medium or high titer (i.e., > 40 GPL or MPL, or > the 99th percentile) on two or more occasions, at least 12 weeks apart, measured by a standardized ELISA.	
**3.** Anti-β2-glycoprotein 1 antibody of IgG or IgM isotype, or both, in serum or plasma (in titers greater than the 99th percentile) present on two or more occasions, at least 12 weeks apart, measured by a standardized ELISA, according to recommended procedures.	

## Pathogenesis of Antiphospholipid syndrome

Though the exact pathogenesis is not known, two possible mechanisms have been described to explain the pathophysiology of APS: antiphospholipid antibodies may alter hemostatic reaction by cross-linking cell surface-bound antigens or aPL may directly trigger cell activation resulting in alterations in expression or production of various molecules [3, 4].

Anti-β2GPI antibodies are known to be one of the major responsible causes of thrombotic events in APS [4, 9, 10]. It has been demonstrated that anti-β2GPI antibodies potentiate thrombosis by binding to β2GPI on cell surfaces activating endothelial cells, monocytes, platelets, neutrophils, fibroblasts, and trophoblasts. Such action induces various pathways, depending on cell type [10, 11].

**Fig. 1:**
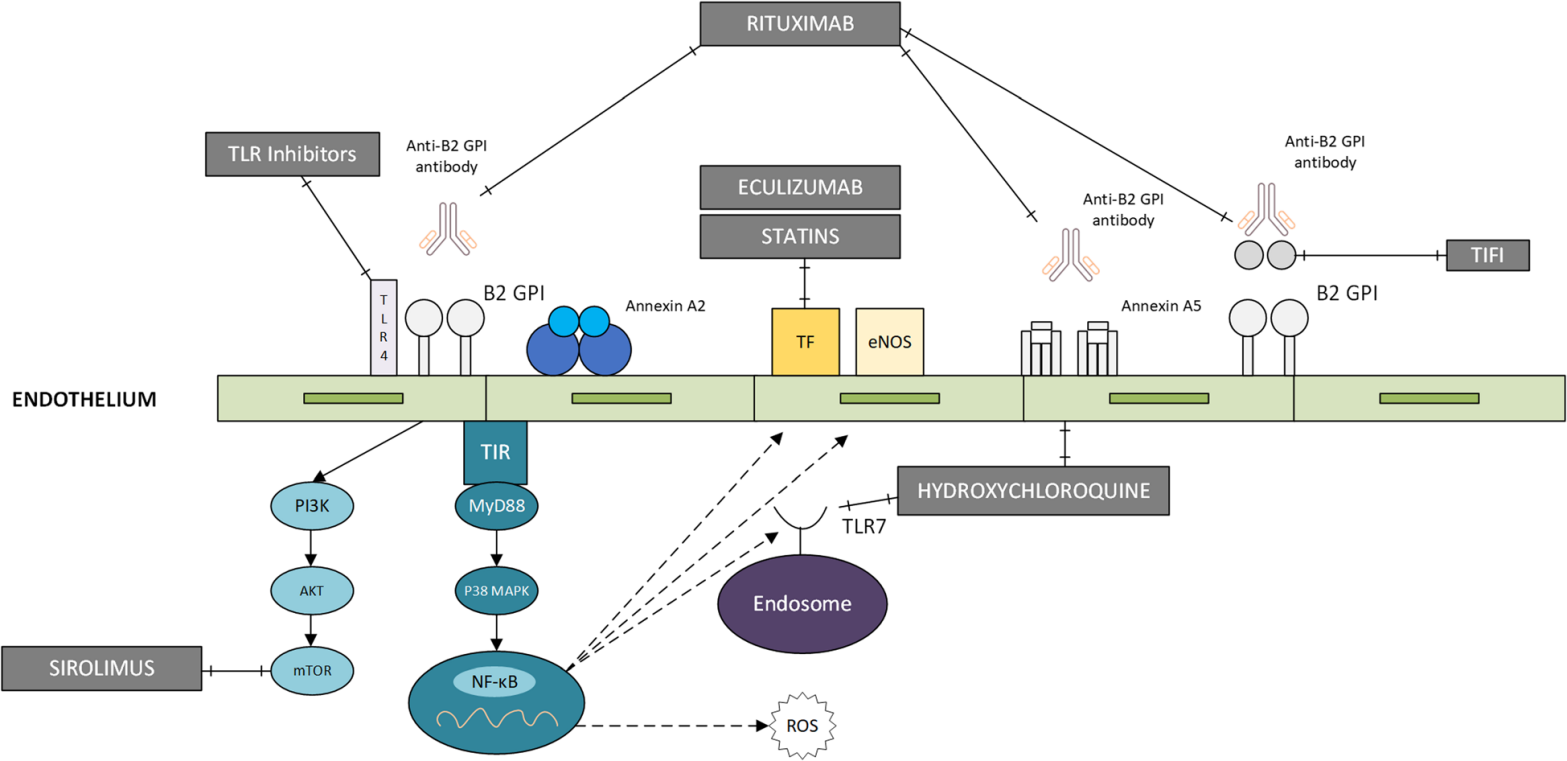
Endothelial cell activation and mechanism action of potential future therapeutics in antiphospholipid syndrome [12]. β2GPI: β2 glycoprotein-I; TLR: toll-like receptor; TF: tissue factor; eNOS: endothelial nitric oxide synthase; mTOR: mammalian target of rapamycin; ROS: reactive oxygen species; NFκB: nuclear factor kappa-light-chain-enhancer of B cells

Recent studies suggest that APS is more related to endothelial cell activation (Fig. 1) rather than antibody-mediated coagulation [5]. Endothelial cell activation through Toll-like receptor (TLR) family as a result of anti-β2GPI antibodies and endothelial cell interaction was first described in 2003 [13]. Allen et al. showed the effect of TLR4 in endothelial cell activation in response to anti-β2GPI antibodies. They also demonstrated that TLR4 signaling consisted of a complex of protein including annexin A2, TLR4, calreticulin, and nucleolin [14]. A recently discovered pathway shows that anti-β2GPI antibodies may cause endothelial cell activation by releasing endothelial cell-derived extracellular vesicles through TLR7 and that these endothelial vesicles may contribute to the activation of unstimulated neighboring endothelial cells by paracrine signaling [15].

Another arterial vascular endothelial related pathology described in APS is intimal hyperplasia. Arterial vasculopathy in APS is a contributor of large artery occlusion rather than thrombosis [16]. It has been recently demonstrated that aPLs activate AKT/ mammalian target of rapamycin (mTOR) pathway in the endothelial cells and cause proliferation of endothelial and vascular smooth muscle actin cells. A correlation between anticardiolipin and anti-β2GPI antibodies titers and mTOR pathway activation degree has also been shown [16].

Platelet activation also has an important effect on thrombus formation in ALP. Anti-β2GPI antibodies bind to β2GPI receptors on platelets and can cause both arterial and venous thrombosis, resulting in increased production of glycoprotein 2b-3a and thromboxane A2 [5, 17].

Other studies demonstrate that neutrophils may also contribute to pathologic clotting, especially venous thrombosis in APS [18, 19]. Neutrophils release neutrophil extracellular traps (NETs), which consist of nucleus originated DNA and histones, as well as cytoplasm derived granule proteins, such as neutrophil elastase and myeloperoxidase. Intrinsic coagulation cascade can be activated by the DNA part of NETs. Proteases in NETs play a role in inactivation of particular anticoagulation factors [18, 20, 21]. Furthermore, histones induce platelets [18, 22]. In addition, APS neutrophils show proinflammatory signature expression related to interferon (IFN)-mediated signaling pathway, cellular defense and intercellular adhesion. Neutrophil adhesion also has an important role in thrombus formation. It has been shown that P-selectin glycoprotein ligand-1 (PSGL-1), which interacts with endothelial selectins in neutrophil rolling, is upregulated in APS. In that same study, PSGL-1 deficiency was demonstrated to prevent antiphospholipid antibody-mediated thrombosis by affecting neutrophil and NETs mediated pathway [23].

In pregnancy, β2GPI is noted to be present on placental trophoblast cells and maternal decidual cells. In vitro studies have shown that aPLs engaging with trophoblast cells can cause proinflammatory cytokine secretion, inhibition of trophoblast migration, increased secretion of trophoblast anti-angiogenic sEng and disruption of trophoblast-endothelial interaction in spiral artery transformation [24].

## Clinical manifestations

The classic clinical presentation of APS is characterized by venous and arterial thrombosis, fetal losses, and thrombocytopenia. Deep venous thrombosis of the lower limbs is the most common clinical subtype of venous thrombosis followed by pulmonary embolism. Although arterial thromboembolic events are less common, it has a higher mortality and morbidity rate as it frequently affects the cerebrovascular bed, usually causing strokes and transient ischemic attacks [6, 9]. Vascular occlusions may present in any form and combination in the same patient with time intervals between vascular events showing variation from weeks to months, or even years [25].

Pregnancy-related complications are one of the hallmarks of APS and include early to late fetal loss, premature birth, and preeclampsia [6].

The most severe life-threatening form of APS is called catastrophic antiphospholipid syndrome (CAPS). Although the prevalence of CAPS is less than 1%, it has high mortality rate due to a rapid onset microthrombosis involving multiple organs resulting in multiorgan failure [26]. Table 2 shows various clinical characteristics of APS related with venous and arterial thrombosis as well as non-thrombotic manifestations.

**Table 2:** Clinical characteristics of antiphospholipid syndrome [6]

**Peripheral thrombosis:**	**Hematological manifestations:**
• Deep venous thrombosis	• Thrombocytopenia
• Superficial thrombophlebitis in legs	• Hemolytic anemia
• Arterial thrombosis in legs	**Gastrointestinal manifestations:**
• Venous thrombosis in arms	• Esophageal ischemia
• Arterial thrombosis in arms	• Mesenteric ischemia
• Subclavian vein thrombosis	• Splenic infarction
• Jugular vein thrombosis	**Osteo-articular manifestations:**
**Neurologic manifestations:**	• Arthralgia
• Migraine	• Arthritis
• Stroke	• Avascular necrosis of bone
• Transient ischemic attack	**Cutaneous manifestations:**
• Epilepsy	• Livedo reticularis
• Multi-infarct dementia	• Ulcers
• Chorea	• Pseudovasculitic lesions
• Acute encephalopathy	• Digital gangrene
**Pulmonary manifestations:**	• Cutaneous necrosis
• Pulmonary embolism	**Obstetric manifestations:**
• Pulmonary hypertension	• Preeclampsia
• Pulmonary microthrombosis	• Eclampsia
**Cardiac manifestations:**	• Abruptio placentae
• Valve thickening/dysfunction	**Fetal manifestations:**
• Myocardial infarction	• Early fetal losses (< 10 weeks)
• Angina	• Late fetal losses (≥10 weeks)
• Myocardiopathy	• Live births
• Vegetations	• Premature births
• Coronary by-pass rethrombosis	
**Renal manifestations:**	
• Glomerular thrombosis	
• Renal infraction	
• Renal artery thrombosis	
• Renal vein thrombosis	

Recently, arterial and venous thromboses have been described as severe complications of coronavirus disease 2019 (COVID-19), caused by the novel severe acute respiratory system coronavirus (SARS-CoV-2). The underlying mechanism of COVID-19-related coagulopathy remains unclear [27–29]. However, studies have shown elevated aPL levels in a considerable number of patients with severe COVID-19 [27–31]. Elevated NET levels from neutrophils have also been reported in severe COVID-19 patients [29]. Infection-induced aPL is usually transient and may disappear in a couple of weeks [27–31]. Although the clinical significance of this associated transient increase in aPL levels has not been well-defined, these transient findings may play a role in the development of thrombotic events and may lead to APS in particularly severe COVID-19 cases. Thus far, there have been no reports of APS or APS-like disease affecting the eye that was deemed to be secondary to or associated to COVID-19 infection.

## Ocular manifestations

Antiphospholipid syndrome can affect any part of the eye including the anterior and posterior segments, as well as visual pathways in the central nervous system [11]. Therefore, ocular involvement of APS may show up with a wide range of clinical manifestations and symptoms. Patients with ocular involvement typically present with visual symptoms such as monocular or binocular blurring of vision, amaurosis fugax, transient diplopia and transient visual field losses [3, 11, 32]. Headaches and migraine-like visual symptoms have also been reported [3]. Table 3 demonstrates the various documented ocular manifestations of APS.

**Table 3:** Ocular Manifestations of Antiphospholipid Syndrome

**Anterior Segment Manifestations:**	
• Conjunctivitis sicca	
• Conjunctival vascular telangiectasias and microaneurysms	
• Punctate epithelial keratopathy	
• Limbal keratitis	
• Iritis	
• Episcleritis	
• Scleritis	
**Posterior Segment Manifestations:**	
• Retinal microvascular occlusions	
• Central retinal vein occlusion	
• Central retinal artery occlusion	
• Branched retinal vein occlusion	
• Branched retinal artery occlusion	
• Choroidal infarction	
• Cilioretinal infarction	
• Vitritis	
• Retinal vasculitis	
• Serpiginous like choroidopathy	
• Frosted branch angiitis	
• Posterior scleritis	
**Neuro-ophthalmologic Manifestations:**	
• Ischemic optic neuropathy	
• Ophthalmoplegia	
• Ischemic infarcts of visual pathway	

### Anterior segment

Anterior segment involvement of APS is relatively rare compared to posterior segment diseases. Clinical findings seen in the anterior segment include conjunctivitis sicca, conjunctival vascular telangiectasias and microaneurysms, punctate epithelial keratopathy, and limbal keratitis [11, 32, 33]. Anterior uveitis associated with retinal vasculitis, episcleritis, and scleritis have been described as clinical findings of APS [32, 34].

### Posterior segment

**Fig. 2:**
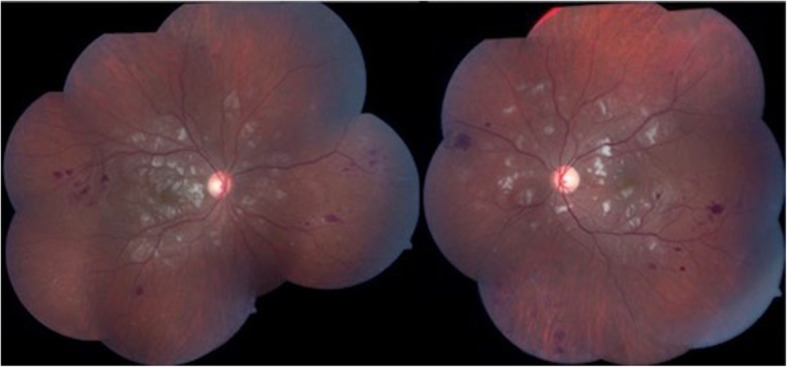
Composite color fundus photographs of both eyes demonstrate cotton wool spots in the posterior pole and multiple retinal hemorrhages along with vascular sheathing in the peripheral retina of a patient with primary antiphospholipid syndrome

Antiphospholipid syndrome more commonly presents with posterior segment involvement and is typically associated with vaso-occlusive conditions. Common retinal findings seen in APS include venous tortuosity, retinal hemorrhages, microaneurysms, and cotton-wool spots, the latter of which is most likely attributable to microvascular occlusion (Fig. 2) [3, 33, 35]. The use of spectral domain optical coherence tomography (SD-OCT) has shown retinal ischemia (paracentral acute middle maculopathy, PAMM) in patients with primary APS as hyperreflective, band-like lesions located in the inner or outer retinal layers [36, 37]. Optical coherence tomography angiography (OCTA) has likewise demonstrated evidence of superficial and deep capillary deficits with signal attenuation artifacts [36].

**Fig. 3:**
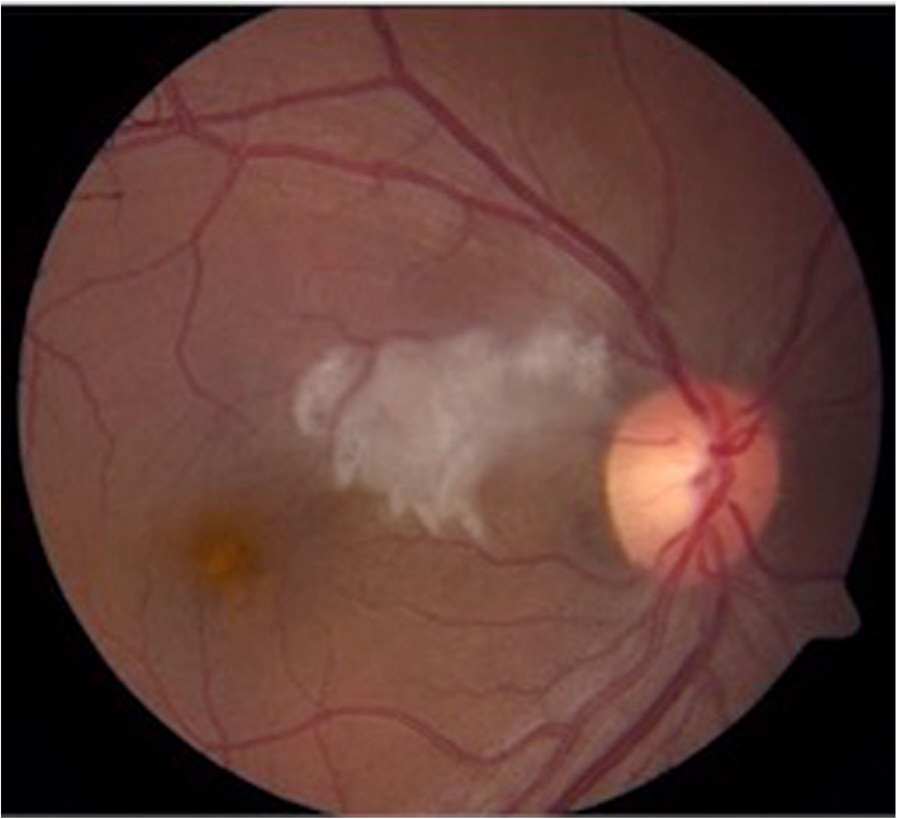
Color fundus photography of the right eye shows retinal whitening involving the superior papillomacular bundle corresponding to a cilioretinal arterial occlusion in a patient with primary antiphospholipid syndrome

Other vaso-occlusive pathologies that are also common in APS include: central retinal vein occlusion (CRVO), central retinal artery occlusion (CRAO), branched retinal vein occlusion (BRVO), and branched retinal artery occlusion (BRAO) [11, 32]. Left untreated, vaso-occlusive retinopathies may lead to several complications such as neovascularization, vitreous hemorrhage, neovascular glaucoma, and tractional retinal detachment [3]. Choroidal infarction and cilioretinal infarction can also be seen in APS (Fig. 3) [11, 32].

Instead of retinal venous occlusions occurring at arterial/venous crossings as seen in atherosclerosis-related vasculopathy, APS-related venous occlusions may be seen in multiple areas in the same eye [3, 33, 35]. Similarly, retinal arterial occlusions do not usually occur at bifurcations as seen in embolic situations [3, 33, 35].

In retinal vein occlusion, the presence of aPLs has been reported as higher compared with population-based controls [38–41]. A recent systemic review and meta-analysis assessing the risk of retinal vein occlusion showed a significant association between aPLs and risk of developing retinal vein occlusion (OR = 5.18, 95% CI = [3.37, 7.95]) [42]. The British Committee for Standards in Haematology released guidelines in 2012 suggesting that all patients with unprovoked episodes of venous occlusion without other underlying systemic of local causes be tested for aPLs after a sufficient period of removing anticoagulation treatment [43]. Though venous occlusion involving the eye was not specifically mentioned, given the higher prevalence of aPLs in patients with retinal vein occlusion [38–41] and increased risk of developing of retinal vein occlusion in the presence of aPLs [42, 44, 45] that has been reported in several studies, aPLs are potential targets to detect and measured in patients with newly diagnosed retinal vein occlusion, especially in patients without any known systemic underlying conditions such as hypertension, diabetes, hyperlipidemia, and cardiovascular disease.

**Fig. 4:**
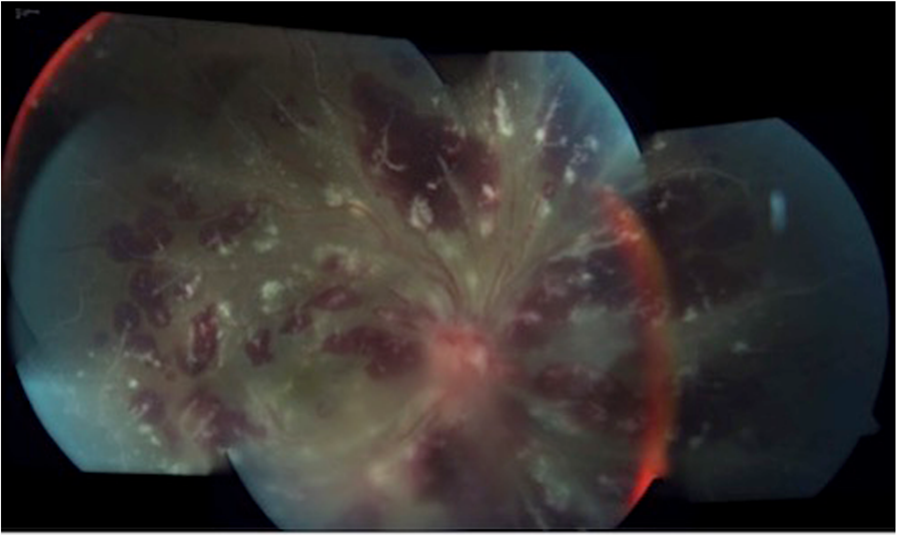
Composite color fundus photograph of the right eye shows widespread retinal vasculitis with perivascular exudates, optic disc swelling, macular edema and diffuse retinal hemorrhages, characterizing frosted branch angiitis in a patient with secondary antiphospholipid syndrome from systemic lupus erythematosus

In a descriptive study of 13 patients positive for aCL, retinal vasculitis, vitritis, and posterior scleritis have been seen despite no other diagnosable etiology [34]. Serpiginous-like choroidopathy due to both retinal and choroidal vaso-occlusion accompanied by vitritis and vasculitis was also described in APS [46]. Wood and et al. presented a case of iritis in the right eye and bilateral vitritis with diffuse retinal peri-phlebitis resembling frosted branch angiitis in a patient with primary APS, All ocular findings resolved with oral prednisone treatment [47] Fig. 4 depicted a frosted branch angiitis in a patient with antiphospholipid syndrome.

### Neuro-ophthalmologic manifestations

APS can present in a wide range of neuro-ophthalmologic disorders such as non-arteritic or arteritic ischemic optic neuropathy, extraocular motility disorders, and central nervous system (CNS) infarctions along the visual pathway [3, 11, 32].

Optic disc findings due to non-arteritic and arteritic ischemic optic neuropathy are common neuro-ophthalmologic presentations in APS patients [32]. Retrobulbar optic neuropathy with normal fundus examination accompanied by decreased visual acuity and visual field defects have been shown in primary APS patients [48].

Ocular motility disorders due to extraocular muscle nerve palsies have been reported in APS patients in the settings of idiopathic intracranial hypertension and cerebral sinus thrombosis [49, 50]. Superior ophthalmic vein thrombosis is another pathology that can also cause ophthalmoplegia as well as proptosis in APS patients [51].

## Treatment

Prevention of aPL-induced coagulation is the main goal in the treatment of thrombotic events in APS. Initial therapy for patients with venous thrombosis consists of unfractionated or low-molecular-weight heparin (LMWH) for 5 days followed by anticoagulation therapy with warfarin [9, 12]. The international normalized ratio (INR) target ranges between 2.0 and 3.0. High intensity anticoagulation, in which the INR target is between 3 and 4, has not shown superiority in terms of preventing recurrent thrombosis in patients with APS [52, 53].

Oral anticoagulation therapy with a target INR of 2.0–3.0 is also recommended for patients with arterial non-cerebral events [1, 17]. Higher intensity anticoagulation is preferred in some centers because some studies have shown that moderate intensity anticoagulation was not able to prevent recurrences in arterial thrombosis [9, 12]. Low-dose aspirin therapy with moderate intensity anticoagulation therapy is suggested for APS patients with cerebral events [17, 54].

Life-long anticoagulation is essential in APS patients who have thrombotic event history [1, 12].

Regarding APS associated with pure ophthalmic manifestations however, there are no current guidelines nor any therapeutic actions suggested other than for preventing subsequent events. Furthermore, systemic anticoagulation, particularly with warfarin, has been linked to increased risk of subsequent retinal vein and arterial occlusion, even at therapeutic dosages [55–57].

### Novel anticoagulants

Recently, direct oral anticoagulants (DOACs) have been developed. These direct FXa inhibitors include rivaroxaban, apixaban and edoxaban and a direct thrombin inhibitor named dabigatran [1, 12, 58, 59]. All of these DOACs are reversible, competitive, and dose dependent and have not shown inferiority to Vitamin K antagonists (VKA) in terms of secondary prevention of venous thrombosis and stroke in patients with nonvalvular atrial fibrillation [12, 59]. Furthermore, they have some advantages over VKAs because they are used as a fixed dose, do not need monitoring, and show fewer drug interactions. However, there are ongoing clinical trials comparing DOACs and warfarin in APS patients and we have limited data regarding the overall safety DOACs in treatment compared to VKAs [1, 12, 58].

### Hydroxychloroquine

Hydroxychloroquine (HCQ) is an antimalarial drug that is typically used in SLE patients which has anti-inflammatory as well as anti-thrombotic effects [12, 17, 26]. It has been shown that HCQ has anti-thrombotic effects based on inhibition of glycoprotein IIb/IIIa (GPIIb/IIIa) expression on aPL activated platelets [60], preventing aPL-β2GPI-phospholipid formation, and aPL-mediated Annexin A5 shield disruption (Fig. 1) [61–63]. Furthermore, HCQ is known to have immunomodulatory effects including prevention of Toll-like receptor 3 (TLR3), Toll-like receptor 7 (TLR7) (Fig. 1) and Toll-like receptor 9 (TLR9) activation as well as reduction in IFN signature and aPL titers [64–66]. On the basis of anti-thrombotic and immunomodulatory properties, HCQ can be considered as an adjunctive therapy for APS patients who have recurrent thrombotic events despite being on adequate anticoagulation therapy [5, 12, 58]. However, further clinical studies are needed supporting the clinical antithrombotic effects of HCQ in patients with APS.

### Statins

Statins, also known as hydroxymethylglutaryl coenzyme A (HMG-CoA) reductase inhibitors, are a type of cholesterol lowering agent. In addition to a lipid-lowering effect, statins have demonstrated anti-thrombotic and anti-inflammatory properties such as inhibition of tissue factor production in endothelial cells and prevention of anti-β2GPI antibody-mediated endothelial adherence (Fig. 1) [67, 68]. Fluvastatin treatment has been proposed to decrease proinflammatory and prothrombotic mediators in aPL positive patients and to inhibit several mediators in monocytes that may be pro-thrombotic in APS patients [69, 70].

### Rituximab

Rituximab is a chimeric monoclonal antibody against CD 20 expressed on B cells that was originally developed for the treatment of non-Hodgkin’s B-cell lymphoma. In the last several years, B-cell targeting therapies have been approved for the treatment of various autoimmune diseases [71]. Anti-CD20 monoclonal therapy has been shown to decrease aPL titers and prevent new thrombotic events (Fig. 1) [72]. Moreover, in a non-randomized prospective pilot study, rituximab was found to be effective in the treatment of non-criteria aPL manifestations in a persistently aPL positive patients [73]. It has also been a safe and effective therapeutic option for patients with refractory catastrophic APS [74].

### Eculizumab

Eculizumab is a humanized monoclonal antibody against complement C5 that inhibits C5 cleavage into C5a and C5b, thus preventing membrane attack complex formation. It is currently used for paroxysmal nocturnal hemoglobinuria [75, 76]. C5 is a strong proinflammatory, chemotactic, and anaphylatoxic molecule that also shows prothrombotic effect by regulating many mediators from numerous cells [77]. It induces tissue factor expression on endothelial cells, neutrophils, and monocytes [77–79]. Figure 1 depicted mechanism action of eculizumab in endothelial cells. Eculizumab has been used in treatment of refractory CAPS [76, 80–84] and aPL-mediated acute thrombotic microangiopathy after renal transplantations [76, 83, 85].

### mTOR inhibition

Inhibition of mammalian target of rapamycin (mTOR) pathway can prevent APS-related vasculopathy by inhibiting proliferation of endothelial and vascular smooth muscle cells (Fig. 1) [16]. Sirolimus, an mTOR inhibitor, reduced the development of intimal hyperplasia and showed better graft survival in patients with APS who underwent renal transplantation [86]. It has also been shown that anti-β2GPI results in mTOR activation as well as tissue factor and Interleukin-8 (IL-8) expression in monocytes [87]. Based on these findings, mTOR inhibition can be considered as a useful therapeutic option in the future in terms of preventing aPL-mediated thrombosis and inflammation in APS patients.

### Future therapeutic targets

TIFI is a 20-amino acid peptide derived from cytomegalovirus, which shows similarities with the PL-binding site of β2GPI [12, 88]. TIFI inhibits the binding of β2GPI to human endothelial cells and murine monocytes in vitro in a dose-dependent manner (Fig. 1). TIFI has also been demonstrated to reverse aPL-induced thrombosis in mice [89]. It was also shown to inhibit binding of monoclonal human β2GPI to human trophoblast in vitro as well as to reverse aPL-induced fetal loss and growth retardation in pregnant mice [90]. Similarly, recombinant β2GPI -DI abrogates aPL-induced thrombus formation and inhibits production of vascular cell adhesion molecule-1 (VCAM-1) in aortic endothelial cells and macrophage tissue factor in mice [91].

Inhibitors of intracellular signaling pathways in APS pathogenesis are also promising therapeutic targets as inhibition of nuclear factor kappa-light-chain-enhancer of B cells (NFκB) reduces proinflammatory and prothrombotic features of aPL as well as reverses aPL-induced thrombosis in mice [92, 93]. Moreover, inhibition of Toll-like receptor 4 (TLR4) (Fig. 1) by an intracellular domain of TLR4 prevents anti-β2GPI/β2GPI-induced tissue factor (TF) and tumor necrosis factor-alpha (TNF-α) productions, which may contribute to thrombus formation [94].

## Conclusion

Antiphospholipid syndrome is characterized by both arterial and venous thrombosis, fetal loss, and thrombocytopenia in the presence of antiphospholipid bodies [1, 2]. It may present in various clinical forms with different thrombotic events as well as non-thrombotic events [6]. Ocular involvements can occur in 15–88% of patients with APS and clinical findings may vary depending on the affected part of the eye including: anterior segment, posterior segment and the visual pathway along the central nervous system [3, 11]. Since APS is common and has a variety of ocular findings, early recognition of the ocular manifestations related with APS syndrome is crucial in order to prevent future thrombotic events and life-threatening morbidities.

The mechanism of the APS syndrome has not been fully understood, but recent studies have shown that aPL-induced cell activation plays an important role in the pathogenesis of APS more so than antibody-mediated coagulation. Antiphospholipid antibodies, especially anti-β2GPI antibodies, contribute to thrombosis formation by activating different cell types including endothelial cells, monocytes, platelets, neutrophils, fibroblasts, and trophoblasts, leading to induction of various pathways [10, 11]. Although anticoagulation therapy is still the standard treatment method in the management of APS, better understanding of the mechanism and identifying more specific tools in the pathogenesis of APS may provide target-specific therapy and better control of disease in patients who are refractory to standard therapy.

## Abbreviations

APS: Antiphospholipid syndrome
aPL: Antiphospholipid antibodies
LA: Lupus anticoagulant
SLE: Systemic lupus erythematosus
aCL: Anti-cardiolipin antibodies
anti-β2GPI: Anti-β2 glycoprotein-I
mTOR: Mammalian target of rapamycin
TLR: Toll-like receptor
NETs: Neutrophils release neutrophil extracellular traps
IFN: Interferon
PSGL-1: P-selectin glycoprotein ligand-1
CAPS: Catastrophic antiphospholipid syndrome
COVID-19: Coronavirus disease 2019
SARS-CoV-2: Severe acute respiratory system coronavirus
SD-OCT: Spectral domain optical coherence tomography
CRVO: Central retinal vein occlusion
CRAO: Central retinal artery occlusion
BRVO: Branched retinal vein occlusion
BRAO: Branched retinal artery occlusion
LMWH: Low-molecular-weight heparin
INR: International normalized ratio
DOACs: Direct oral anticoagulants
VKA: Vitamin K antagonists
HCQ: Hydroxychloroquine
GPIIb/IIIa: Glycoprotein IIb/IIIa
HMG-CoA: Hydroxymethylglutaryl coenzyme A
IL-8: Interleukin-8
VCAM-1: Vascular cell adhesion molecule-1
NFκB: Nuclear factor kappa-light-chain-enhancer of B cells
TF: Tissue factor
TNF-α: Tumor necrosis factor-alpha
